# The relationship between mobile phone dependence and academic burnout in Chinese college students: a moderated mediator model

**DOI:** 10.3389/fpsyt.2024.1382264

**Published:** 2024-05-17

**Authors:** Na Li, Linxi Fu, Hewen Yang, Wanting Zhao, Xingbo Wang, Yingchun Yan, Yangyang Fu

**Affiliations:** School of Mental Health, Jining Medical University, Jining, China

**Keywords:** academic burnout, Chinese college students, love, mobile phone dependence, study engagement

## Abstract

**Objective:**

The aim of this study was to examine the correlation between the level of mobile phone dependence among college students and their experience of academic burnout. Additionally, the study sought to explore the potential mediating effect of study engagement and the moderating role of love.

**Methods:**

During October and December 2023, a cross-sectional study measuring mobile phone dependence, academic burnout, and study engagement among Chinese college students, using the UtrechtWork Engagement Scale-student (UWES-S), College Student Mobile Phone Dependence Questionnaire (CSMPDQ), and Academic Burnout Questionnaire (ABQ). To examine the hypothesis of mediating and moderating effect, SPSS PROCESS was utilized.

**Results:**

The predictive effect of mobile phone dependence on academic burnout was significant (*β* = 0.410, *t* = 14.236, *p* < 0.001), and the predictive effect of mobile phone dependence on academic burnout remained significant when the mediating variable study engagement was introduced (*β* = 0.308, *t* = 10.288, *p* < 0.001), mobile phone dependence had a significant predictive effect on study engagement (*β* = -0.292, *t* = -11.639, *p* < 0.001), and study engagement had a significant positive predictive effect on academic burnout (*β* = -0.270, *t* = -9.028, *p* < 0.001). Love significantly negatively predicted study engagement (*β* = -0.564, *t* = -9.641, *p* < 0.001); and the interaction term for mobile phone dependence and love was significant (*β* = -0.211, *t* = -3.688, *p* < 0.001), indicating a significant moderating effect of love between mobile phone dependence and study engagement.

**Conclusion:**

Mobile phones among college students has been found to have a direct correlation with academic burnout. It can also indirectly contribute to academic burnout by diminishing levels of academic engagement. This indirect relationship is further influenced by love. These findings can help researchers and educators better understand the underlying mechanisms between smartphone dependence and learning burnout in undergraduates.

## 1 Introduction

Academic burnout among college students refers to a diminished sense of self-assurance in effectively managing academic obstacles, resulting in a pervasive negative psychological disposition towards educational institutions, scholarly pursuits, and specific courses (1, 2). Empirical evidence indicates that a substantial proportion of college students, comprising 61.63%, experience academic burnout, with 37.5% facing more severe manifestations of this condition. Conversely, only 5.3% of college students remain unaffected by academic burnout, while the remaining students exhibit varying degrees of this phenomenon (3). Individuals exhibiting mobile phone dependence may experience a range of negative consequences, including physical discomfort (4), heightened aggression (5), impulsive tendencies (6), impaired inhibitory control (6), diminished sleep quality (7–9), reduced concentration abilities, academic underperformance (10), and strained interpersonal relationships (11). Furthermore, research suggests that excessive cell phone use can detrimentally affect mental health, particularly in adolescents, leading to heightened levels of anxiety, depression, and stress, as well as influencing impulsive behaviors and personality traits (4, 9, 12). Alternatively, by examining the outcomes of cross-sectional and longitudinal research, it is feasible to ascertain a potential correlation between mobile phone addiction and depression among individuals (13). Moreover, the adverse psychological state experienced by college students can manifest itself through a pivotal measure known as academic burnout, which profoundly influences the quality of their studies as well as their overall physical and mental well-being (14).

### 1.1 The relationship between mobile phone dependence and academic burnout

The progress in mobile phone technology has significantly enhanced convenience in daily life. However, it has also resulted in a prevalent reliance on mobile phones. This phenomenon encompasses problematic behaviors such as excessive mobile phone usage, an inability to regulate the duration of usage, anxiety upon separation from the device, and adverse repercussions in domains such as academics and interpersonal communication. Some research studies have alternatively labeled this issue as mobile phone dependence or problematic mobile phone use (15, 16). The issue of mobile phone dependence has gained significant attention in current research within the domains of mental disorders, psychology, and social behavior. Extensive studies have consistently demonstrated a correlation between mobile phone dependence and various negative outcomes, including academic performance, concentration difficulties, interpersonal challenges, and a range of psychological issues (17–19). Moreover, the detrimental effects of mobile phone dependence are particularly pronounced among college students (20–22). A strong association between mobile phone dependence and various risk factors in individuals, with academic concerns, particularly academic burnout, emerging as the most prominent risk factor (23, 24). In light of these findings, it can be inferred that mobile phone dependence among college students significantly impacts their academic burnout. Furthermore, the study posits a hypothesis (H1) suggesting a positive correlation between mobile phone dependence and academic burnout.

### 1.2 The mediating effect of study engagement

Learning engagement, which refers to the level of intensity and emotional involvement that students dedicate to the process of acquiring and implementing learning tasks, can be fostered through positive emotions (affective engagement), goal-oriented thinking (cognitive engagement), and active participation (behavioral engagement) (25, 26). The influences on learning engagement can be classified into two overarching categories: individual factors (such as demographic and psychometric variables) (27–30) and environmental factors (31, 32) (including family and school variables), as a result of the interplay between these factors. Initial research efforts were primarily dedicated to investigating the correlation between academic burnout and academic engagement at the level of individual psychometric variables (28, 30, 33, 34). Subsequent studies have provided evidence indicating that the extent of burnout exerts a detrimental influence on engagement and serves as a negative predictor of academic burnout (35). In comparison to other factors, the association between academic engagement and outcomes related to academic burnout was found to be more substantial (36). Nevertheless, the precise mechanism underlying the relationship between mobile phone dependence and academic burnout in terms of learning engagement remains ambiguous. Consequently, we propose the hypothesis that learning engagement serves as a mediator between mobile phone addiction and academic burnout (H2).

### 1.3 The moderating effects of love

In modern higher education institutions, students encounter a multitude of challenges and vexations stemming from diverse origins such as academic demands, interpersonal dynamics, and adaptability, thereby leading to heightened psychological distress among young adults (37–39). Furthermore, the ubiquity of mobile phones exacerbates the adverse outcomes associated with excessive reliance on these devices. Based on the theory of personality development stages in the context of romantic relationships, individuals between the ages of 18 and 25, commonly referred to as adolescents, seek emotional support to navigate adversities such as conflict and stress by establishing intimate connections through love (40). Nevertheless, certain studies have indicated that college students who are engaged in love exhibit psychological traits characterized by exclusivity, volatility, and impulsivity, thereby potentially compromising their levels of self-control and rationality (41), which in turn may impact their academic engagement. To date, there is a lack of empirical research on the impact of love on college students’ level of academic engagement. Consequently, we put forth the hypothesis that love might exert a moderating influence on the association between mobile phone dependency and academic engagement (H3), and investigate the role of love in the connection between mobile phone dependency and academic engagement.

**Figure 1:**
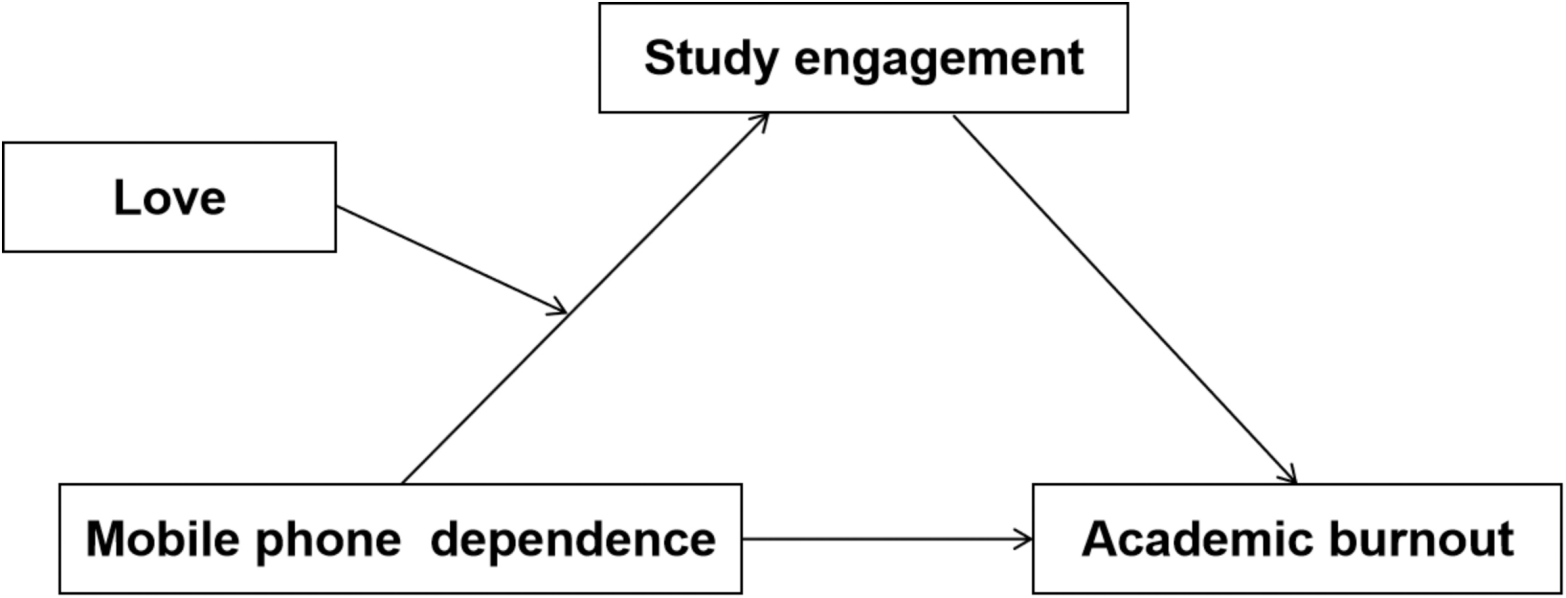
Hypothetical model.

This study aims to examine the correlation between mobile phone dependence and academic burnout among college students, utilizing them as the primary research participants. Additionally, the study seeks to explore the potential mediating effect of study engagement and the moderating effect of love. This study attempts to construct a mediated model (**Figure 1**). The ultimate objective is to expand the existing knowledge on mobile phone dependence and academic burnout, with the intention of mitigating academic burnout issues among students and enhancing their academic achievements.

## 2 Methods

### 2.1 Study design and data collection

This study employed a randomized questionnaire survey to collect data from undergraduate college students in Shandong Province, utilizing the Questionnaire Star platform. The research protocol obtained approval from the Ethics Committee of Jining Medical University. To participate in this study, each individual was obligated to complete an informed consent form. Informed consent and parental/guardian consent were obtained for participants under the age of 18. The volunteers did not receive any monetary incentives throughout the duration of the trial. A total of 1100 subjects obtained during the period of October to December 2023 were analyzed in this study. Ninety-four questionnaires were excluded due to insufficient response time (less than 200 seconds), presence of randomized regular responses, uniform selection of options for all questions, and consistent responses to reverse and forward questions. This resulted in 1006 valid questionnaires, yielding an effective recovery rate of 91.45%. The sample consisted of 482 male students (47.9%) and 524 female students (52.1%).

### 2.2 Research tools

#### 2.2.1 College student mobile phone dependence questionnaire

The study employed the Mobile Phone Dependence Scale for College Students, which was developed by Wang (2013) (42). This scale includes five dimensions: conflict, salience, withdrawal, persistence, and technology. It consists of a total of 20 questions, such as “Mobile phones are more important than clothes and food,” “I feel uneasy without my mobile phone,” and “I’d rather lose my wallet than my mobile phone,” among others. The scale is assessed using a five-point scale ranging from 1 (hardly at all) to 5 (always), with higher scores indicating a stronger inclination towards mobile phone dependence. The criteria for mobile phone dependence tendency were established as a total score of ≥ 70, while a total score of ≥ 80 was used to define mobile phone dependence syndrome. The questionnaire exhibited a commendable overall consistency coefficient of 0.948, indicating satisfactory construct validity and acceptable internal consistency. These test findings offer substantiation for the reliability and validity of the scale.

#### 2.2.2 Academic burnout scale

The Academic Burnout Questionnaire devised by Wu et al. (2007) was employed for this study (43). The scale consisted of 16 items, including statements such as “I am capable of dedicating myself to my academic pursuits with vigor,” “I have been experiencing a sense of emptiness recently and am unsure of how to address it,” and “I am struggling academically and feel inclined to abandon my efforts.” These items were categorized into three dimensions: physical and mental fatigue, disengagement from academic activities, and poor academic performance. These items are scored on a 5-point Likert scale ranging from 1 (completely disagree) to 5 (completely agree). The total score ranges from 16 to 80, with higher scores indicating greater academic burnout. The overall consistency coefficient for this instrument was determined to be 0.907. The findings of the analysis indicated that the scale exhibited favorable structural validity and satisfactory internal consistency.

#### 2.2.3 UtrechtWork engagement scale-student

In this study, the utilization of the UtrechtWork Engagement Scale-student (UWES-S) developed by Liao (2011) was implemented. This scale comprises three distinct dimensions: behavioral input, cognitive input, and emotional input (44). It comprises a total of 20 inquiries, including statements such as “The usual holiday will not relax study,” “Spare time will not relax study,” and “After class will be self-review,” among others. The assessment instrument was evaluated using a five-point Likert scale, ranging from “not at all” to “completely”. Higher scores on this scale indicate higher levels of learning engagement. The scale demonstrated a high internal consistency, with an alpha coefficient of 0.944. The findings of the test indicated favorable structural validity for the scale.

### 2.3 Statistical analysis

Descriptive statistics, independent samples t-test, one-way ANOVA, repeated measures ANOVA, and product-difference correlation analysis were conducted using SPSS 22.0, as well as mediating effects tests and moderated mediating effects tests using Model 4 and Model 7 of Hayes’ PROCESS macro program.

## 3 Results

### 3.1 Common method bias test

The presence of common method bias in the data was assessed through the application of Harman’s one-way factor analysis. The findings revealed the existence of 18 factors with eigenroot values surpassing 1, and the initial factor accounted for 29.24% of the explained variance (which falls below the 40% threshold). Consequently, it can be concluded that this study did not exhibit any significant common method bias.

### 3.2 Correlations among mobile phone dependence, learning engagement, academic burnout and love

Pearson product-moment correlation was employed to examine the association between the four variables, and the findings are displayed in **Table 1**. The findings indicate a notable association among mobile phone dependence, academic engagement, academic burnout, and love. A positive and significant correlation exists between mobile phone dependence and academic burnout, thereby validating Hypothesis 1. A negative and significant correlation is observed between academic engagement and academic burnout. A positive and significant correlation is found between love and academic burnout. Mobile phone dependence exhibits a negative and significant correlation with academic engagement. Mobile phone dependence is also significantly negatively correlated with academic engagement. A noteworthy inverse correlation is observed between mobile phone dependency and academic engagement.

**Table 1:** Descriptive statistics and correlations among variables.

	M	SD	1	2	3	4
1.Mobile phone dependence	3.018	1.006	1			
2.Study engagement	3.355	0.900	-0.378**	1		
3.Academic burnout	3.269	0.639	0.410**	-0.386**	1	
4.Love	0.260	0.439	0.175**	-0.352**	0.129**	1

^**^ *p*<0.01.

### 3.3 Mediating effect of learning engagement between mobile phone dependence and learning burnout

Mediation test Model 4, developed by Hayes based on the SPSS macro program PROCESS, uses the non-parametric percentage Bootstrap method with bias correction to extract an estimated 95% confidence interval repeatedly for 5000 times. In this analysis, mobile phone dependence was considered as the independent variable, academic burnout as the dependent variable, academic engagement as the mediator variable, love as the moderating variable. Independent variables in the final multivariate regression model were selected using a stepwise procedure and P-values of regression coefficients were calculated by permutation tests. The corresponding outcomes are presented in **Table 2**. Mobile phone dependence can significantly and positively predict academic burnout (*β* = 0.410, *t* = 14.236, *p* < 0.001), and the regression coefficient of mobile phone dependence on academic burnout becomes smaller after adding the mediator variable of study engagement, but it is still significant (before adding the mediator variable, *β* = 0.410, *p* < 0.001, and after adding it, *β* = 0.308, *p* < 0.001), which indicates that study engagement has an important role in the partial mediating role between mobile phone dependence and academic burnout. The bias-corrected Bootstrap test showed the results in **Table 3**: the mediating effect of study engagement was significant, with a direct effect value of 0.196 and a 95% confidence interval of [0.154, 0.237]; and an indirect effect value of 0.065 and a 95% confidence interval of [0.047, 0.084]. That is, study engagement plays a partial mediating role between mobile phone dependence and learning burnout, and Hypothesis 2 is established.

**Table 2:** Intermediary model testing.

Variable	Model 1		Model 2		Model 3	
	Academic burnout		Study engagement		Academic burnout	
	β	t	β	t	β	t
Mobile phone dependence	0.410	14.236***	-0.378	-12.932***	0.308	10.288***
Study engagement					-0.270	-9.028***
R	0.410		0.378		0.480	
R^2^	0.168		0.143		0.231	
F	202.674***		167.225***		150.215***	

***p<0.001.

**Table 3:** Bootstrap based mediation effect test.

Effect type	Pathway	Effect	BootSE	BootLLCI	BootULCI
Direct effect	Mobile phone dependence−Academic burnout	0.1955	0.0208	0.1535	0.2371
Indirect effect	Mobile phone dependence−Study engagement−Academic burnout	0.0648	0.0093	0.0471	0.0838
Total effect		0.2603	0.0210	0.2190	0.3020

### 3.4 The moderating effect of love on the relationship between mobile phone dependence and learning engagement

**Figure 2:**
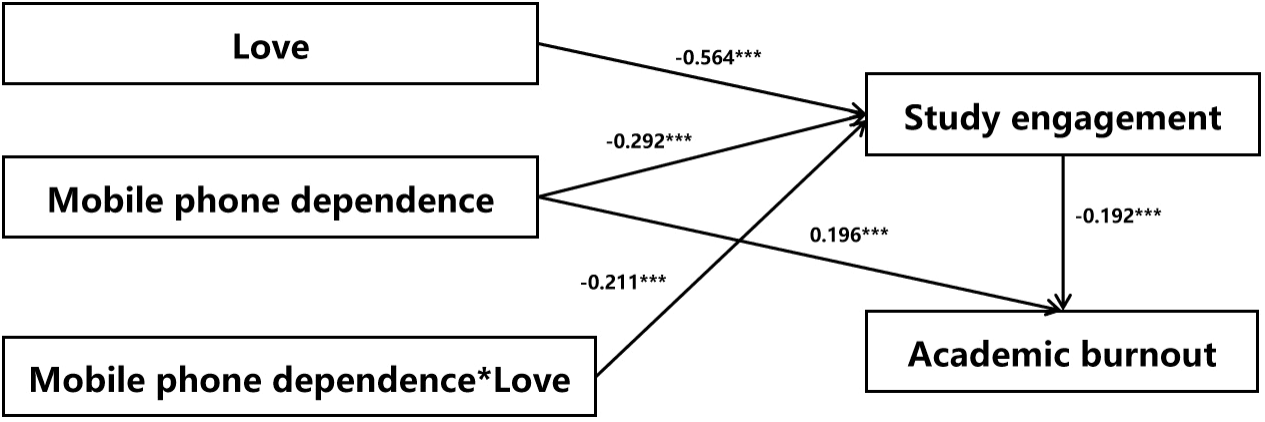
Intermediary model. ***p < 0.001.

The moderating role of love in the relationship between mobile phone dependence and learning engagement was analyzed using Model 7 of process v4.1 macro program for data processing in SPSS software. In order to reducing non-essential collinearity and improve the interpretation of the results, the independent variables were centered as suggested by Cohen and Hayes. And we calculated the product term between the centered independent variable and the centered moderator as the interaction term. The results showed that mobile phone dependence had a significant negative predictive effect on study engagement (*β* = -0.292, *t* = -11.639, *p* < 0.001); love had a significant negative predictive effect on study engagement (*β* = -0.564, *t* = -9.641, *p* < 0.001); the interaction term mobile phone dependence and love mobile phone dependence * love had a significant predictive effect on study engagement (*β* = - 0.211, *t* = -3.688, *p* < 0.001). It suggests that the mediating effect of mobile phone dependence on academic burnout via academic engagement was moderated by love (**Table 4**; **Figure 2**).

**Table 4:** Moderated mediation model tests.

Variable	Model 1			Model 2		
	Study engagement			Academic burnout		
	B	SE	t	B	SE	t
Mobile phone dependence	-0.292	0.025	-11.639***	0.196	0.019	10.288***
Study engagement				-0.192	0.021	-9.028***
Love	-0.564	0.059	-9.641***			
Mobile phone dependence * Love	-0.211	0.057	-3.688***			
R	0.487			0.480		
R^2^	0.237			0.231		
F	103.952***			150.215***		

***p<0.001.

The results of Bootstrap analysis showed that love had a significant moderating effect in the effect of mobile phone dependence on learning engagement. The mediating effect value of study engagement without love is 0.045 (BootSE = 0.008), and its 95% confidence interval is [0.031, 0.062], the mediating effect is significant, and the mediating effect value of study engagement with love is 0.086 (BootSE = 0.015), and its 95% confidence interval is [0.059, 0.116], the mediating effect is significant (**Table 5**). The effect size of the mediating effect is larger with love than without love, and the 95% confidence interval of index indicator is [0.016, 0.066], which indicates that love moderates the mediating effect of learning engagement between mobile phone dependence and learning burnout (**Table 6**), and hypothesis 3 is established.

**Table 5:** The mediating effect of love.

Mediated variable	Effect	BootSE	BootLLCI	BootULCI
*Non-love*	0.045	0.008	0.031	0.062
*love*	0.086	0.015	0.059	0.116

**Table 6:** Index.

	Index	BootSE	BootLLCI	BootULCI
Love	0.040	0.013	0.016	0.066

**Figure 3:**
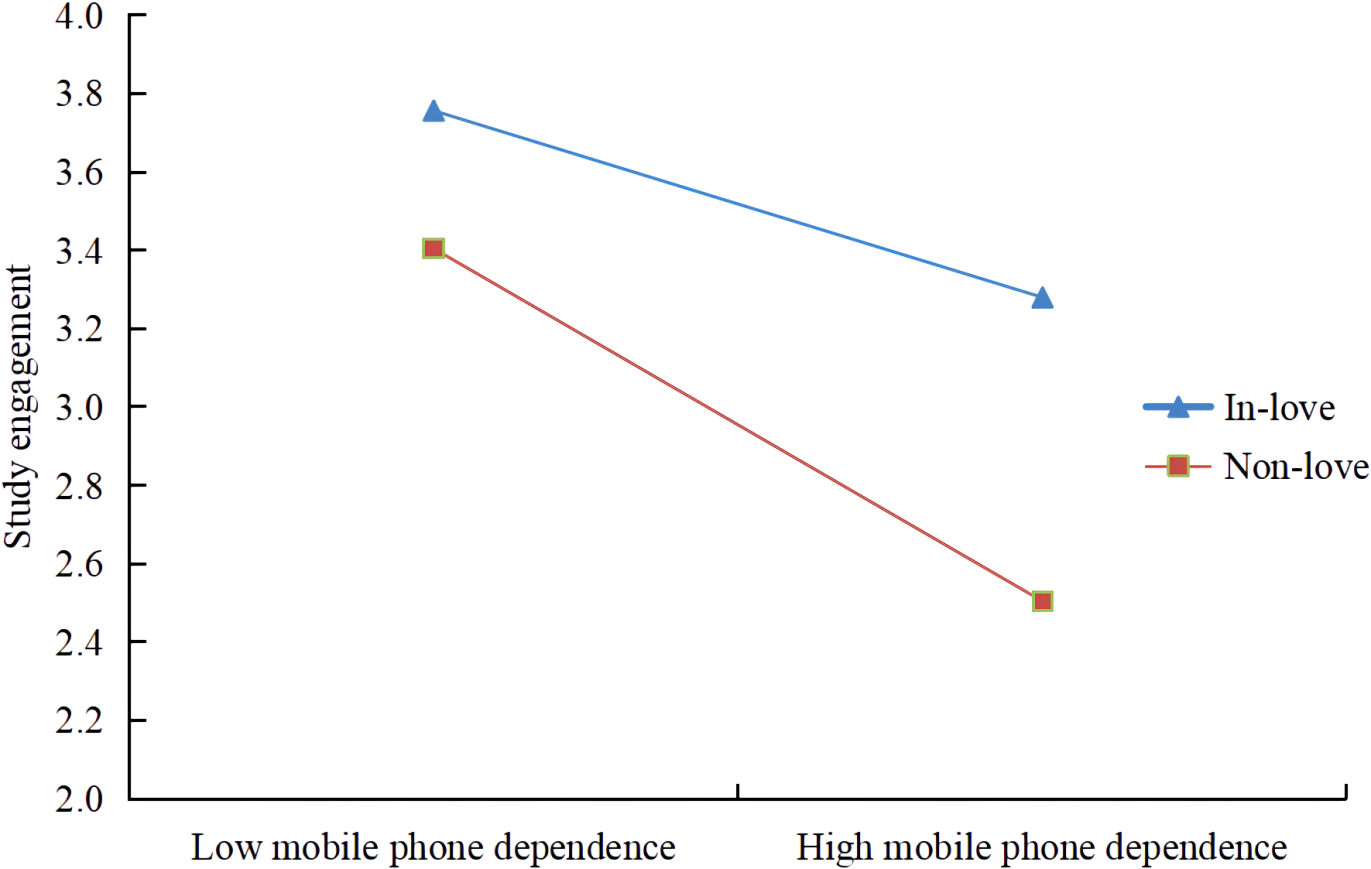
Effects of mobile phone dependence on learning engagement in love and non-love.

The simple slope test shows that mobile phone dependence has a significant negative predictive effect on study engagement in the absence of love, and the predictive effect of mobile phone dependence on study engagement is more pronounced with love (**Table 7**; **Figure 3**). This finding suggests that the detrimental impact of mobile phone dependence on study engagement is progressively amplified when compared to individuals without love. Additionally, it demonstrates that in the context of high mobile phone dependence, love has the potential to mitigate the adverse predictive influence on study engagement.

**Table 7:** The affect of love.

Mediated variable	Effect	se	t	p	LLCI	ULCI
*Non-love*	-0.237	0.029	-8.126	0.000	-0.294	-0.180
*Love*	-0.448	0.049	-9.117	0.000	-0.544	-0.351

## 4 Discussion

This study developed a moderating mediator model to investigate the impact of mobile phone dependence on academic burnout, and to examine whether this impact is influenced by the presence of love. The results shed light on the connection between academic burnout, mobile phone dependence, and love, thus offering a valuable theoretical foundation for further exploration and extension of research on the relationship between mobile phone dependence and academic burnout. Furthermore, this study offers empirical evidence and theoretical direction for the prevention and intervention of academic burnout among students.

### 4.1 The mediating role of learning engagement

The current study utilized regression analysis to confirm the predictive influence of college students’ mobile phone dependence behavior on academic burnout. Specifically, it was found that mobile phone dependence partially and positively predicts academic burnout, mediated by academic engagement. These findings support hypotheses 1 and 2, aligning with previous research (30, 45). The results suggest a negative correlation between the level of mobile phone dependence and the level of commitment to learning, as well as a positive correlation between mobile phone dependence and academic burnout. This relationship is attributed to the impact of mobile phone dependence on reducing self-control and increasing negative emotions among college students (24, 46). Previous scholars have established a comprehensive model theory and behaviorist theory of mobile phone dependence, which can help explain this phenomenon (23). Additionally, the “participation-identification” model of academic engagement is also relevant in understanding this relationship (47).

The self-control ability of college students is weakened by their dependence on mobile phones (48). The path model theory of mobile phone dependence posits that the initial path of mobile phone use is characterized as “impulsive”, wherein college students excessively utilize mobile phones due to a lack of control over their behavior and emotional regulation (23). This impulsivity directly impacts an individual’s commitment to learning, consequently resulting in negative emotions associated with the learning process (49). The participation-identification model of learning engagement posits that college students’ positive behavioral engagement during the learning process can foster their interest in learning and subsequently enhance their overall engagement (47). Conversely, the detrimental impact of mobile phone dependence on students’ learning emotions significantly diminishes their interest in learning, undermines their dedication to academic pursuits, and contributes to the occurrence of academic burnout.

The utilization of mobile phones among college students has been found to contribute to heightened negative emotions. This phenomenon aligns with the behaviorism theory, which posits that individual addiction is a gradual progression characterized by escalating emotional responses (23). Initially, individuals experience satisfaction from using mobile phones, leading to a gradual development of obsession. Consequently, when unable to access their devices, individuals experience amplified negative emotions. Based on the “participation-identification” model, enhanced study engagement necessitates heightened “identification”, referring to the positive emotional encounter individuals derive from educational pursuits, while excessive cell phone usage is likely to amplify negative emotions among individuals. The excessive utilization of mobile devices is anticipated to amplify the adverse affective states experienced by individuals, particularly college students. These negative emotions are expected to impede their emotional involvement during study sessions, thereby hindering the acquisition of positive emotional encounters while studying. Consequently, this diminished level of learning engagement is likely to culminate in academic burnout.

### 4.2 The moderating effect of love

This study aimed to construct a mediation model to examine the moderating role of love in the mediating effect of “mobile phone dependence→study engagement→academic burnout”. The findings indicated a significant moderating effect of relationship, primarily influencing the initial phase of the mediating process. The findings of the survey indicate that the impact of love on study engagement is more pronounced among individuals with high levels of mobile phone dependence compared to those with low levels of mobile phone dependence. Specifically, students who are in love and exhibit high mobile phone dependence experience a lesser degree of influence on their study engagement due to their mobile phone dependence. However, the level of academic engagement of students in love is higher in both the low mobile phone dependence and high mobile phone dependence states. According to previous literature, the emergence of this phenomenon may be related to three factors: 1) the emotional support brought by falling in love (50); 2) the sense of self-worth in the process of falling in love (51); and 3) falling in love enhances self-esteem and self-confidence (52, 53).

Social-emotional support is a crucial aspect of relationships, as evidenced by pertinent research. Although social-emotional support may not have a direct impact on academic burnout, it indirectly fosters academic engagement through the influence of love (54). Furthermore, it is noteworthy that love tend to elicit a greater provision of emotional support. Furthermore, scholarly sources have substantiated the notion that love hold significant significance in an individual’s life and personal growth. Notably, research indicates a positive association in love during adolescence and heightened feelings of self-worth (55, 56). Moreover, it has been observed that individuals are more inclined to attain a sense of self-worth within the context of love. Research in the field of social psychology reveals that individuals in healthy romantic partnerships experience feelings of care and support from their partners, leading to a heightened sense of intimacy and security. This, in turn, contributes to an increase in self-esteem and self-confidence. The emotional support, enhanced sense of self-worth, and elevated self-confidence derived from such relationships have a beneficial impact on college students, as they alleviate the stress associated with academic pursuits and daily life, mitigate the adverse consequences of excessive reliance on mobile phones, and enhance levels of academic engagement.

This study aimed to develop a mediation model incorporating moderating effects to elucidate the mediating and moderating roles of study engagement and love in the impact of mobile phone dependence on academic burnout among college students. On one hand, this study contributes to the understanding of the underlying causes and mechanisms of academic burnout among college students, particularly highlighting the detrimental effects of mobile phone dependence. On the other hand, it offers valuable research support for college mental health educators, urging them to prioritize students with mobile phone dependence and concentrate on enhancing their self-control, modifying their mobile phone usage patterns, and fostering increased study engagement as a means to alleviate academic burnout.

## 5 Limitations

This study examined the impact of mobile phone dependence on the severity of academic burnout in relation to love and study engagement, utilizing a substantial sample size. However, it is important to acknowledge that there are certain limitations inherent in the research process. This study employed a sample consisting solely of college students to investigate the correlation and underlying mechanisms linking mobile phone dependence and academic burnout. However, additional research is warranted to ascertain the generalizability of these findings to junior and senior high school students. Furthermore, it is important to note that this study solely utilized path analysis and cross-sectional analysis, thereby limiting its ability to establish a causal relationship between the independent variables and the dependent variable. Consequently, future investigations should incorporate multiple variables and employ a longitudinal research design to establish the causal relationship and elucidate the intricate mechanisms underlying the association between college students’ mobile phone dependence and academic burnout.

## 6 Conclusions and recommendations

This study developed a moderated mediation model to investigate the impact of mobile phone dependence on academic burnout of college students, as well as the potential involvement of study engagement and love in mediating this relationship. The findings of the research indicate a significant correlation between mobile phone dependence and academic burnout of college students. Mobile phone dependence is shown to have a direct impact on these capabilities, as well as an indirect influence through its effects on study engagement. Additionally, the deleterious effects of mobile phone dependence on academic engagement were progressively magnified for the romantically involved population compared to those who were not in love. Based on these results, the following recommendations are proposed:

Firstly, artificial intelligence-assisted screening methods, such as chatbots and large-scale social media data analysis (57), are employed to detect mobile phone dependence among college students in a timely manner. This program is designed to identify at-risk individuals and implement effective interventions to address the issue.

Secondly, enhancing the school’s guidance and supervision mechanisms to mitigate students’ reliance on mobile phone, fostering positive habits in both academic and personal spheres, redirecting attention from mobile devices to academic pursuits, fortifying resistance against negative influences, and bolstering academic dedication.

Thirdly, guiding students in the judicious allocation of time to diminish mobile phone usage, thereby promoting a consistent daily routine and ensuring students’ sustained energy levels in the classroom to prevent academic burnout.

Fourthly, schools should enhance thematic education on the concept of love and prioritize providing support and guidance to students who are excessively reliant on mobile phones while in romantic relationships.

## Data availability statement

The original contributions presented in the study are included in the article/supplementary material. Further inquiries can be directed to the corresponding authors.

## Author contributions

NL: Writing – review & editing, Writing – original draft, Project administration, Formal analysis, Data curation. LF: Writing – original draft, Visualization, Formal analysis. HY: Writing – review & editing, Methodology, Investigation. WZ: Writing – review & editing, Visualization, Methodology. XW: Writing – original draft, Visualization, Formal analysis. YY: Writing – review & editing, Validation, Supervision, Funding acquisition. YF: Writing – review & editing, Supervision, Funding acquisition, Conceptualization.
